# Enantioselective dioxytosylation of styrenes using lactate-based chiral hypervalent iodine(III)

**DOI:** 10.3762/bjoc.14.53

**Published:** 2018-03-20

**Authors:** Morifumi Fujita, Koki Miura, Takashi Sugimura

**Affiliations:** 1 Graduate School of Material Science, University of Hyogo, Kohto, Kamigori, Hyogo 678-1297, Japan

**Keywords:** 1,2-difunctionalization of alkenes, enantioselective synthesis, hypervalent iodine, oxidation

## Abstract

A series of optically active hypervalent iodine(III) reagents prepared from the corresponding (*R*)-2-(2-iodophenoxy)propanoate derivative was employed for the asymmetric dioxytosylation of styrene and its derivatives. The electrophilic addition of the hypervalent iodine(III) compound toward styrene proceeded with high enantioface selectivity to give 1-aryl-1,2-di(tosyloxy)ethane with an enantiomeric excess of 70–96% of the (*S*)-isomer.

## Findings

**Scheme 1:**
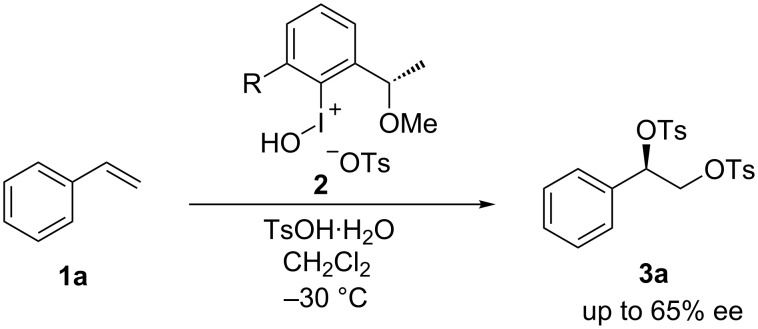
Enantioselective dioxytosylation of styrene as a seminal example.

Hypervalent aryl-λ^3^-iodanes have been widely used for metal-free oxidation with high selectivity in organic synthesis [1–3]. The reactivity of an aryl-λ^3^-iodane is controlled by the electronic and steric properties of the aryl group and the heteroatomic ligand coordinated to the iodine atom. Optically active hypervalent iodine compounds contain chiral ligands or chiral aryl groups. Several types of optically active hypervalent iodine reagents and catalysts have been developed for highly stereocontrolled oxidative transformations [4–14]. The enantioselective vicinal difunctionalization of alkenes constitutes one type of attractive transformation achieved by chiral hypervalent iodine compounds. As a seminal example in this field, Wirth et al. [15–17] reported the dioxytosylation of styrene (**1a**, Scheme 1). Chiral hypervalent iodine reagents **2** bearing a 1-methoxyethyl side chain were used for enantiocontrol of the dioxytosylation, and the maximum enantiomeric excess (ee) of the product **3a** reached 65%. Despite recent rapid progress in the field of asymmetric oxidation achieved by chiral hypervalent iodine compounds, there has been no subsequent examination of dioxytosylation, which can be used as a standard reaction for comparing the enantiocontrolling ability of chiral hypervalent iodine reagents.

**Figure 1:**
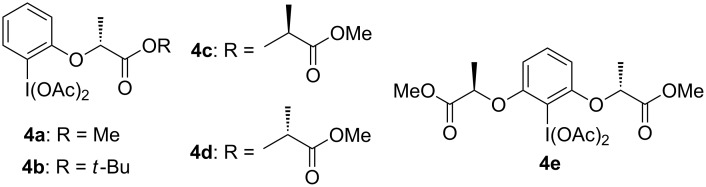
Series of lactate-based hypervalent iodine reagents.

The design of chiral hypervalent iodine reagents using a lactate motif has been employed for several types of oxidation reaction since we first reported this procedure [18]. Enantioselective oxidative transformations include the dearomatization of phenols [19–24], α-functionalization of carbonyl compounds [25–29], and vicinal difunctionalization of alkenes [18,30–50]. Here, the efficiency of the lactate-based chiral hypervalent iodine reagents **4a–e** (Figure 1) was assessed using the dioxytosylation of styrenes as a reference reaction.

A series of lactate-derived aryl-λ^3^-iodanes **4a**–**e** was used for the oxidation of styrenes **1** in the presence of *p*-toluenesulfonic acid (TsOH) in dichloromethane. The reaction proceeded at −50 °C to give the 1,2-dioxytosylated product **3** and the rearranged product **5**. The yields of **3** and **5** were determined by ^1^H NMR using an internal standard. The ee of **3** was determined by chiral HPLC analysis. The results for the yields and ee are summarized in Table 1.

**Table 1:** Enantioselective dioxytosylation of styrenes **1** using aryl-λ^3^-iodanes **4**.^a^

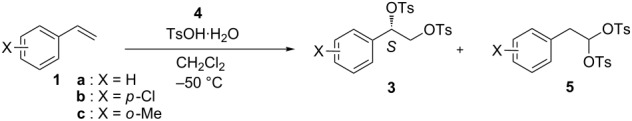					
				
			Yield (%)^b^		
		
Entry	Substrate	Reagent	**3**	**5**	ee of **3** (%)^c,d^

1	**1a** (X = H)	**4a**	53	15	70 (*S*)

2	**1a** (X = H)	**4b**	49	16	80 (*S*)

3	**1a** (X = H)	**4c**	41	14	78 (*S*)

4	**1a** (X = H)	**4d**	41	22	70 (*S*)

5	**1a** (X = H)	**4e**	80	20	92 (*S*)

6	**1b** (X = *p*-Cl)^e^	**4a**	63	6	70

7	**1b** (X = *p*-Cl)^e^	**4b**	46	5	76

8	**1b** (X = *p*-Cl)^e^	**4e**	79	5	90

9	**1c** (X = *o*-Me)	**4a**	7	34	79

10	**1c** (X = *o*-Me)	**4e**	10	35	96

^a^The reaction was carried out at −50 °C in dichloromethane containing **4** (47 mM), TsOH (86 mM), and **1** (43 mM) for 4 h. ^b^The yield was determined by ^1^H NMR using an internal standard. ^c^The ee was determined by chiral HPLC using a Daicel CHIRALPAK AD column (ø 4.6 mm × 250 mm). ^d^Preferential configuration of product **3**. The absolute stereochemistry of **3b** and **3c** was not determined. ^e^The reaction was carried out for 20 h.

The reaction of styrene (**1a**) with **4a** gave the 1,2-dioxytosylated product **3a** with 70% ee of the (*S*)-isomer (Table 1, entry 1). An ee of equal to or greater than 70% was also achieved in the reactions with the other lactate-based reagents **4b**–**e** (Table 1, entries 2–5). The reaction with the 2,6-bis(lactate)aryl reagent **4e** provided a high ee of 92%. The reactions of *p*-chlorostyrene (**1b**) gave **3b** with a similar ee, and the ratios of **3** to **5** (**3b** to **5b**) were higher than those in the reaction of **1a** (Table 1, entries 6–8). In the reactions of *o*-methylstyrene (**1c**), the ee of the 1,2-dioxytosylated product **3c** was slightly higher than those of **3a** and **3b**, but the regioselectivity for **3c** over **5c** was poor (Table 1, entries 9 and 10).

**Scheme 2:**
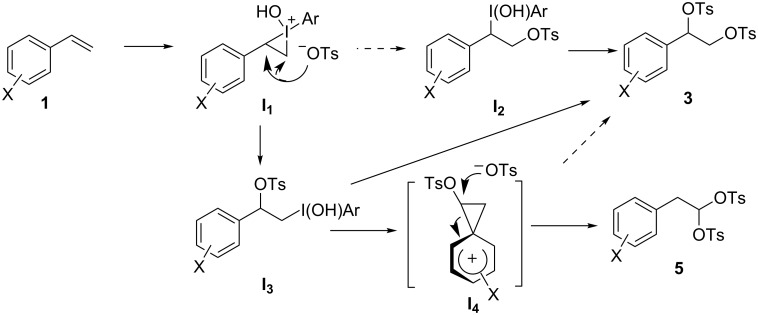
Plausible pathways in dioxytosylation of styrenes.

Scheme 2 illustrates possible reaction pathways that lead to **3** and the achiral byproduct **5**. The treatment of (diacetoxyiodo)benzene with TsOH readily gives Koser’s reagent [PhI(OH)OTs] [51], which has a higher electrophilicity toward the carbon–carbon double bond in **1**. The dioxytosylation of alkenes with Koser’s reagent was found to proceed via an S_N_2 reaction of a cyclic intermediate such as **I_1_**, judging from the *syn* selectivity of the dioxytosylation [52–53]. The attack of the tosylate ion on **I_1_** possibly takes place at the benzylic position or at the methylene carbon atom. The positive charge of **I_1_** may be stabilized by the aryl group and localized at the benzylic position. This may allow the preferential formation of **I_3_** from **I_1_**. If **I_2_** was the major intermediate in the pathway leading to **3**, the stereochemical purity of **3** would have decreased owing to the facile elimination of the iodonium group [54] at the benzylic position of **I_2_** (S_N_1). The high enantiomeric ratio of **3** can be rationalized via a preference for the **I_1_**→**I_3_**→**3** pathway over the **I_1_**→**I_2_**→**3** pathway. The product ratio of **3** to **5** was affected by the ring substituent in styrenes **1**: the electron-withdrawing chloro substituent in **1b** increased the amount of **3**, whereas the electron-donating methyl substituent in **1c** decreased the amount of **3**. An electron-donating aryl group increases the rate of participation of the aryl group (**I_3_**→**I_4_**). In other words, a reaction pathway that bifurcates from **I_3_** to **3** and **5** agrees well with the regioselectivity for **3** over **5** observed for the substituted styrenes. The phenonium cation intermediate **I_4_** contains two reaction sites on the ethylene bridge. Electron donation due to the lone pair on the oxygen atom of the internal tosyloxy group may weaken the bond between the tosyloxy-bonded carbon and the quaternary carbon in **I_4_**.

The reaction of styrene with **4a**–**e** preferentially gave (*S*)-**3**, which forms via an electrophilic addition of the iodane toward the *Si* face of styrene, followed by an S_N_2 reaction with the tosylate ion. If an S_N_1 mechanism were involved in the oxytosylation of **I_1_**, the enantiomeric ratio of **3** would decrease owing to the planar structure of the benzylic cation. Thus, the tosylate ion may act as an effective nucleophile for the S_N_2 reaction of **I_1_**. The stereoface-differentiation in the dioxytosylation reaction using the lactate-derived aryl-λ^3^-iodanes is similar to that in preceding reactions [14], which include the diacetoxylation [38–39,50] and diamination [30,49] of styrene.

In summary, the reaction of styrenes with lactate-derived aryl-λ^3^-iodanes gave the dioxytosylated product with an ee of 70–96%.

## Supporting Information

File 1 Experimental procedures, characterization data, and copies of ^1^H and ^13^C NMR spectra are available.
