# Supplementary material for: Enantioselective dioxytosylation of styrenes using lactate-based chiral hypervalent iodine(III)
Source: Beilstein J Org Chem. 2018 Mar 20;14:659–63. doi: 10.3762/bjoc.14.53 (PMC5870148; doi:10.3762/bjoc.14.53)
Supplement: File 1 — Experimental procedures, characterization data, and copies of 1H and 13C NMR spectra are available. [file Beilstein_J_Org_Chem-14-659-s001.pdf]

Supporting Information

for

Enantioselective dioxytosylation of styrenes using lactate-based chiral hypervalent iodine(III)

Morifumi Fujita*, Koki Miura and Takashi Sugimura

Address: Graduate School of Material Science, University of Hyogo, Kohto, Kamigori, Hyogo 678-1297, Japan

Email: Morifumi Fujita* - fuji@sci.u-hyogo.ac.jp

* Corresponding author

Experimental procedures, characterization data, and copies of ^{1}H and ^{13}C NMR spectra are available

Table of Contents:

General procedure and characterization S2–S6
NMR spectra S7–S14

Experimental section

General. Proton and ^{13}C NMR spectra were measured on a JEOL ECA-600 spectrometer as solutions in $CDCl_3$. Proton NMR spectra were recorded using the residual $CHCl_3$ as an internal reference (7.24 ppm) and ^{13}C NMR using $CDCl_3$ as an internal reference (77.00 ppm). For NMR in CD_2Cl_2, the residual CH_2Cl_2 was employed as an internal reference (5.32 ppm for ^1H NMR and 53.8 ppm for ^{13}C NMR). For mass spectra measurements, a JEOL JMS-T100LC spectrometer was used. Dichloromethane was purified by distillation over CaH_2. Optically active aryl-λ^3-iodanes **4a**, **4b**, and **4e** were prepared as reported previously [S1]. Styrenes were purchased from TCI and used without further purification. The reaction temperature of dioxytosylation of styrene was controlled using a low temperature bath with magnetic stirrer (EYELA, PSL-1800).

(1*R*,1'*S*)-1-(1-(1'-(Methoxycarbonyl)ethoxycarbonyl)ethoxy)-2-iodobenzene (4c'). A benzene solution containing (*R*)-2-(2-iodophenoxy)propanoic acid (99% ee) [S1] (0.725 g, 2.48 mmol) and thionyl chloride (0.25 mL, 3.4 mmol) was refluxed for 2 h. The solution was concentrated in vacuo, and the residue was dropwise added as a solution of dichloromethane (5 mL) to (*S*)-methyl lactate (0.36 mL, 3.7 mmol) and pyridine (0.4 mL). The solution was stirred for 1.5 h, and then quenched by HCl aq. The mixture was extracted with dichloromethane. Purification of the extracts by chromatography (SiO_2; eluent; 25% EtOAc in hexane) gave **4c'** (0.62 g, 1.64 mmol, 66% yield). No diastereomer (**4d'**) was detected by ^1H NMR (600 MHz) and ^{13}C NMR (150 MHz) measurements. This indicates that no epimerization proceed during the esterification. This also agrees with similar esterification with *tert*-butanol giving the precursor of **4b** [S1]. $[\alpha]_D^{20}$ = –27 (c = 1.16, $CHCl_3$); ^1H NMR ($CDCl_3$, 600 MHz) δ 7.76 (d, *J* = 7.6 Hz, 1H), 7.25 (t, *J* = 7.6 Hz, 1H), 6.76 (d, *J* = 7.6 Hz, 1H), 6.71 (t, *J* = 7.6 Hz, 1H), 5.13 (q, *J* = 6.9 Hz, 1H), 4.81 (q, *J* = 6.9 Hz, 1H), 3.72 (s, 3H), 1.73 (d, *J* = 6.9 Hz, 3H), 1.48 (d, *J* = 6.9 Hz, 3H); ^{13}C NMR ($CDCl_3$, 150 MHz) δ 170.94, 170.60, 156.45, 139.71, 129.35, 123.44, 113.41, 87.01, 73.70, 69.09, 52.41, 18.47, 16.77; IR (film) 1752, 1471, 1279, 1191, 1136, 1097 cm^{-1}; HRMS (ESI+) calcd for $C_{13}H_{15}O_5INa$ (M+Na) 400.9862 found 400.9860.

(1*R*,1'*R*)-1-(1-(1'-(Methoxycarbonyl)ethoxycarbonyl)ethoxy)-2-iodobenzene (4d'). A benzene solution containing (*R*)-2-(2-iodophenoxy)propanoic acid (99% ee) (3.1 g, 10.6 mmol) and thionyl chloride (1.1 mL, 15 mmol) was refluxed for 2 h. The solution was concentrated in vacuo, and the residue was dropwise added as a solution of dichloromethane (15 mL) to (*R*)-methyl lactate (1.5 mL, 15 mmol) and pyridine (1.7 mL). The solution was stirred for 1.5 h, and then quenched by HCl aq. The mixture was extracted with dichloromethane. Purification of the extracts by chromatography (SiO_2;

eluent; 20% EtOAc in hexane) gave **4d'** (1.76 g, 4.7 mmol, 45% yield). No diastereomer (**4c'**) was detected by ^{1}H NMR (600 MHz) and ^{13}C NMR (150 MHz) measurements. $[\alpha]_D^{20}$ = +27 (c = 0.92, $CHCl_3$); ^{1}H NMR ($CDCl_3$, 600 MHz) δ 7.77 (d, *J* = 7.6 Hz, 1H), 7.24 (t, *J* = 7.6 Hz, 1H), 6.77 (d, *J* = 7.6 Hz, 1H), 6.72 (t, *J* = 7.6 Hz, 1H), 5.16 (q, *J* = 6.9 Hz, 1H), 4.81 (q, *J* = 6.9 Hz, 1H), 3.69 (s, 3H), 1.74 (d, *J* = 6.9 Hz, 3H), 1.50 (d, *J* = 6.9 Hz, 3H); ^{13}C NMR ($CDCl_3$, 150 MHz) δ 170.93, 170.56, 156.61, 139.76, 129.28, 123.64, 113.98, 87.48, 73.97, 69.19, 52.37, 18.48, 16.83; IR (film) 1752, 1471, 1277, 1191, 1135, 1097 cm^{-1}; HRMS (ESI+) calcd for $C_{13}H_{15}O_5INa$ (M+Na) 400.9862 found 400.9892.

(1*R*,1'*S*)-1-(1-(1'-(Methoxycarbonyl)ethoxycarbonyl)ethoxy)-2-(diacetoxyiodo)benzene (4c). Oxidation of **4c'** by acetic peracid gave **4c** as an oil, which included acetic acid. The compound was used for oxidation without further purification. $[\alpha]_D^{20}$ = –47 (c = 1.01, $CHCl_3$); ^{1}H NMR ($CDCl_3$, 600 MHz) δ 8.09 (d, *J* = 7.6 Hz, 1H), 7.51 (t, *J* = 7.6 Hz, 1H), 7.07 (d, *J* = 7.6 Hz, 1H), 7.02 (t, *J* = 7.6 Hz, 1H), 5.12 (q, *J* = 6.9 Hz, 1H), 4.90 (q, *J* = 6.9 Hz, 1H), 3.71 (s, 3H), 1.93 (s, 6H), 1.70 (d, *J* = 6.9 Hz, 3H), 1.47 (d, *J* = 6.9 Hz, 3H); ^{13}C NMR ($CDCl_3$, 150 MHz) δ 176.63, 170.52, 170.13, 154.35, 137.62, 134.30, 123.53, 113.75, 113.47, 73.93, 69.20, 52.48, 20.32, 18.19, 16.68; HRMS (ESI+) calcd for $C_{14}H_{18}O_6I$ (M–2OAc+OMe) 409.0148 found 409.0110.

(1*R*,1'*R*)-1-(1-(1'-(Methoxycarbonyl)ethoxycarbonyl)ethoxy)-2-(diacetoxyiodo)benzene (4d). Oxidation of **4d'** by acetic peracid gave **4d** as an oil, which included acetic acid. The compound was used for oxidation without further purification. $[\alpha]_D^{20}$ = –23 (c = 2.18, $CHCl_3$); ^{1}H NMR ($CDCl_3$, 600 MHz) δ 8.09 (d, *J* = 7.6 Hz, 1H), 7.49 (t, *J* = 7.6 Hz, 1H), 7.03 (d, *J* = 7.6 Hz, 1H), 7.02 (t, *J* = 7.6 Hz, 1H), 5.13 (q, *J* = 6.9 Hz, 1H), 4.90 (q, *J* = 6.9 Hz, 1H), 3.65 (s, 3H), 1.91 (s, 6H), 1.70 (d, *J* = 6.9 Hz, 3H), 1.46 (d, *J* = 6.9 Hz, 3H); ^{13}C NMR ($CDCl_3$, 150 MHz) δ 176.60, 170.31, 170.10, 154.43, 137.66, 134.14, 123.63, 114.05, 113.68, 74.08, 69.30, 52.37, 20.27, 18.15, 16.67; HRMS (ESI+) calcd for $C_{14}H_{18}O_6I$ (M–2OAc+OMe) 409.0148 found 409.0125.

Typical procedures for determination of product yields and enantiomeric ratios. Optically active aryl-λ^3-iodane **4a** (100 mg, 0.24 mmol) and *p*-toluenesulfonic acid monohydrate (81 mg, 0.43 mmol) was added to dichloromethane (3 mL), and the mixture was stirred for 1 h at room temperature. Then, the mixture was cooled to −50 °C in a low temperature bath (ELELA PSL-1800). To the mixture was added a dichloromethane solution (3 mL) containing styrene (ca. 20 μL, 0.21 mmol) and 4,4-dimethoxybenzophenone (ca. 5 mg) as an internal standard. The solution added was beforehand analyzed by ^{1}H NMR for determining ratio of styrene and the internal standard. After stirred at −50 °C

for 4 h, the solution was lightly concentrated and analyzed by ^{1}H NMR for determining yields of products using the internal standard. Under the conditions, no reaction of 4,4-dimethoxybenzophenone was observed. For determination of enantiomeric ratio, the crude products were purified by SiO_2 column chromatography (eluent: 30% EtOAc in hexane), and then analyzed by chiral HPLC equipped with Daicel Chiralpak AD (∅ 4.6 mm × 250 mm). The sample was detected at 260 nm. Retention time of (*S*)- and (*R*)-**3a** was 19 and 22 min, respectively, when 7:3 hexane/2-propanol was employed as an eluent at flow rate of 1.0 mL/min. Authentic sample of optically active **3a** was prepared by ditosylation of (*R*)-phenylethane-1,2-diol (TCI).

Isolation of 3a. *p*-Toluenesulfonic acid monohydrate (87 mg, 0.46 mmol) was added to dichloromethane solution (4 mL) containing **4a** (100 mg, 0.24 mmol). The mixture was stirred for 1 h at room temperature and then cooled to −50 °C. Styrene (25 μL, 0.26 mmol) was added to the solution at −50 °C. After stirring at −50 °C for 4 h, the mixture was quenched by H_2O, and extracted with dichloromethane. The organic extracts were concentrated in vacuo, and purification by SiO_2 column chromatography (eluent: 30% EtOAc in hexane) gave **3a** (44 mg, 0.099 mmol, 38% yield).

Isolation of 3b. *p*-Toluenesulfonic acid monohydrate (92 mg, 0.48 mmol) was added to dichloromethane solution (4 mL) containing **4a** (106 mg, 0.25 mmol). The mixture was stirred for 1 h at room temperature and then cooled to −50 °C. 4-Chlorostyrene (27 μL, 0.21 mmol) was added to the solution at −50 °C. After stirring at −50 °C for 21 h, the mixture was quenched by H_2O, and extracted with dichloromethane. The organic extracts were concentrated in vacuo, and purification by SiO_2 column chromatography (eluent: 30% EtOAc in hexane) gave **3b** (40 mg, 0.081 mmol, 39% yield).

1-Phenyl-1,2-di(tosyloxy)ethane (3a). Proton NMR data agree well with the reported values [S2]. Enantiomeric ratio was deteremined by chiral HPLC equipped with Daicel Chiralpak AD (∅ 4.6 mm × 250 mm). The sample was detected at 260 nm. Retention time of (*S*)- and (*R*)-**3a** was 19 and 22 min, respectively, when 7:3 hexane/2-propanol was employed as an eluent at flow rate of 1.0 mL/min. An authentic sample of optically active **3a** was prepared by ditosylation of phenylethane-1,2-diol. In literature [S3], enantiomeric analysis of **3a** was carried out using Chiralcel OD as a chiral HPLC column and 9/1 hexane/2-propanol as an eluent. Enantiomeric excess of some samples were analyzed using Chiralcel OD-H (∅ 4.6 mm × 250 mm) as well as Chiralpak AD. Retention time of (*R*)- and (*S*)-**3a** was 22 and 24 min, respectively, when 9:1 hexane/2-propanol was employed as an eluent at flow rate of 1.0 mL/min for chiral HPLC analysis using Chiralcel OD-H. The enantiomeric excess obtained agrees well

with those using Chiralpak AD. Analyses using the AD column resulted in better separation than those using the OD-H column. $[\alpha]_D^{20}$ = +38 (*c* = 0.52, $CHCl_3$) for a sample of 70% ee of *S*-isomer, which was obtained from the reaction with **4a**.

2-Phenyl-1,1-di(tosyloxy)ethane (5a). Proton NMR data agree well with the reported values [S2].

1-(4-Chlorophenyl)-1,2-di(tosyloxy)ethane (3b). ^{1}H NMR (600 MHz, $CDCl_3$) δ 7.60 (d, *J* = 8.2 Hz, 4H), 7.27 (d, *J* = 8.2 Hz, 2H), 7.20 (d, *J* = 8.2 Hz, 2H), 7.18 (d, *J* = 8.2 Hz, 2H), 7.05 (d, *J* = 8.2 Hz, 2H), 5.50 (dd, *J* = 6.9, 4.8 Hz, 1H), 4.17 (dd, *J* = 11.0, 6.9 Hz, 1H), 4.10 (dd, *J* = 11.0, 4.8 Hz, 1H), 2.43 (s, 3H), 2.39 (s, 3H); ^{13}C NMR (150 MHz, $CDCl_3$) δ 145.25, 145.11, 135.41, 133.26, 132.29, 132.19, 129.89, 129.69, 128.89, 128.22, 127.89, 127.87, 79.00, 69.81, 21.67, 21.62; HRMS (ESI+) calcd for $C_{22}H_{21}{}^{35}ClO_6S_2Na$ (M+Na) 503.0366 found 503.0380. $[\alpha]_D^{20}$ = +35 (*c* = 0.60, $CHCl_3$) for a sample of 70% ee, which was obtained from the reaction with **4a**. Enantiomeric ratio was determined by the chiral HPLC equipped with Daicel CHIRALPAK AD (∅ 4.6 mm × 250 mm). Retention time of **3b** was 20 min (major) and 25 min (minor) when 7:3 hexane/2-propanol was employed as an eluent at flow rate of 1.0 mL/min.

2-(4-Chlorophenyl)-1,1-di(tosyloxy)ethane (5b). Mp = 101.7–102.9 °C (white needle); ^{1}H NMR (600 MHz, $CDCl_3$) δ 7.57 (d, *J* = 8.2 Hz, 4H), 7.22 (d, *J* = 8.2 Hz, 4H), 7.06 (d, *J* = 8.2 Hz, 2H), 6.92 (d, *J* = 8.2 Hz, 2H), 6.27 (t, *J* = 6.2 Hz, 1H), 3.09 (d, *J* = 6.2 Hz, 2H), 2.43 (s, 6H); ^{13}C NMR (150 MHz, $CDCl_3$) δ 145.38, 133.51, 132.98, 131.14, 130.83, 129.72, 128.63, 127.87, 99.20, 41.44, 21.70; HRMS (ESI+) calcd for $C_{22}H_{21}{}^{35}ClO_6S_2Na$ (M+Na) 503.0366 found 503.0385.

1-(2-Methylphenyl)-1,2-di(tosyloxy)ethane (3c). ^{1}H NMR (600 MHz, CD_2Cl_2) δ 7.65 (d, *J* = 8.2 Hz, 2H), 7.60 (d, *J* = 8.2 Hz, 2H), 7.34 (d, *J* = 8.2 Hz, 2H), 7.21 (d, *J* = 8.2 Hz, 2H), 7.19–7.13 (m, 2H), 7.07 (d, *J* = 8.2 Hz, 2H), 5.79 (dd, *J* = 8.2, 3.4 Hz, 1H), 4.17 (dd, *J* = 11.7, 8.2 Hz, 1H), 4.04 (dd, *J* = 11.7, 3.4 Hz, 1H), 2.45 (s, 3H), 2.38 (s, 3H), 2.19 (s, 3H); ^{13}C NMR (150 MHz, CD_2Cl_2) δ 145.82, 145.44, 135.54, 133.76, 132.65, 132.28, 130.91, 130.32, 129.96, 129.47, 128.22, 128.06, 127.18, 126.71, 77.66, 70.07, 21.75, 21.68, 19.00; HRMS (ESI+) calcd for $C_{23}H_{24}O_6S_2Na$ (M+Na) 483.0912 found 483.0885. Enantiomeric ratio was determined by the chiral HPLC equipped with Daicel CHIRALPAK AD (∅ 4.6 mm × 250 mm). Retention time of **7c** was 14 min (major) and 18 min (minor) when 7:3 hexane/2-propanol was employed as an eluent at flow rate of 1.0 mL/min.

2-(2-Methylphenyl)-1,1-di(tosyloxy)ethane (5c). ^{1}H NMR (600 MHz, $CDCl_3$) δ 7.55 (d, *J* = 8.2 Hz, 4H), 7.19 (d, *J* = 8.2 Hz, 4H), 7.08 (t, *J* = 6.9 Hz, 1H), 6.98–6.93 (m, 3H), 6.29 (t, *J* = 6.2 Hz, 1H), 3.15 (d, *J* = 6.2 Hz, 2H), 2.40 (s, 6H), 2.15 (s, 3H); ^{13}C NMR (150 MHz, $CDCl_3$) δ 145.13, 136.87, 132.89, 130.90, 130.84, 130.40, 129.66, 127.86, 127.47, 126.08, 99.06, 39.39, 21.66, 19.36; HRMS (ESI+) calcd for $C_{23}H_{24}O_6S_2Na$ (M+Na) 483.0912 found 483.0888.

Reference

S1. Fujita, M.; Okuno, S.; Lee, H. J.; Sugimura, T.; Okuyama, T. *Tetrahedron Lett*. **2007,** *48*, 8691–8694; Fujita, M.; Yoshida, Y.; Miyata, K.; Wakisaka, A.; Sugimura, T. *Angew. Chem., Int. Ed.* **2010,** *49*, 7068–7071; *Angew. Chem.* **2010,** *122*, 7222–7225.

S2. Koser, G. F.; Rebrovic, L.; Wettach, R. H. *J. Org. Chem*. **1981**, *46*, 4324–4326; Rebrovic, L.; Koser, G. F. *J. Org. Chem*. **1984**, *49*, 2462–2472.

S3. Hirt, U.H.; Spingler, B.; Wirth, T. *J. Org. Chem*. **1998,** *63*, 7674–7679; Hirt, U. H.; Schuster, M. F. H.; French, A. N.; Wiest, O. G.; Wirth, T. *Eur. J. Org. Chem*. **2001,** 1569–1579.

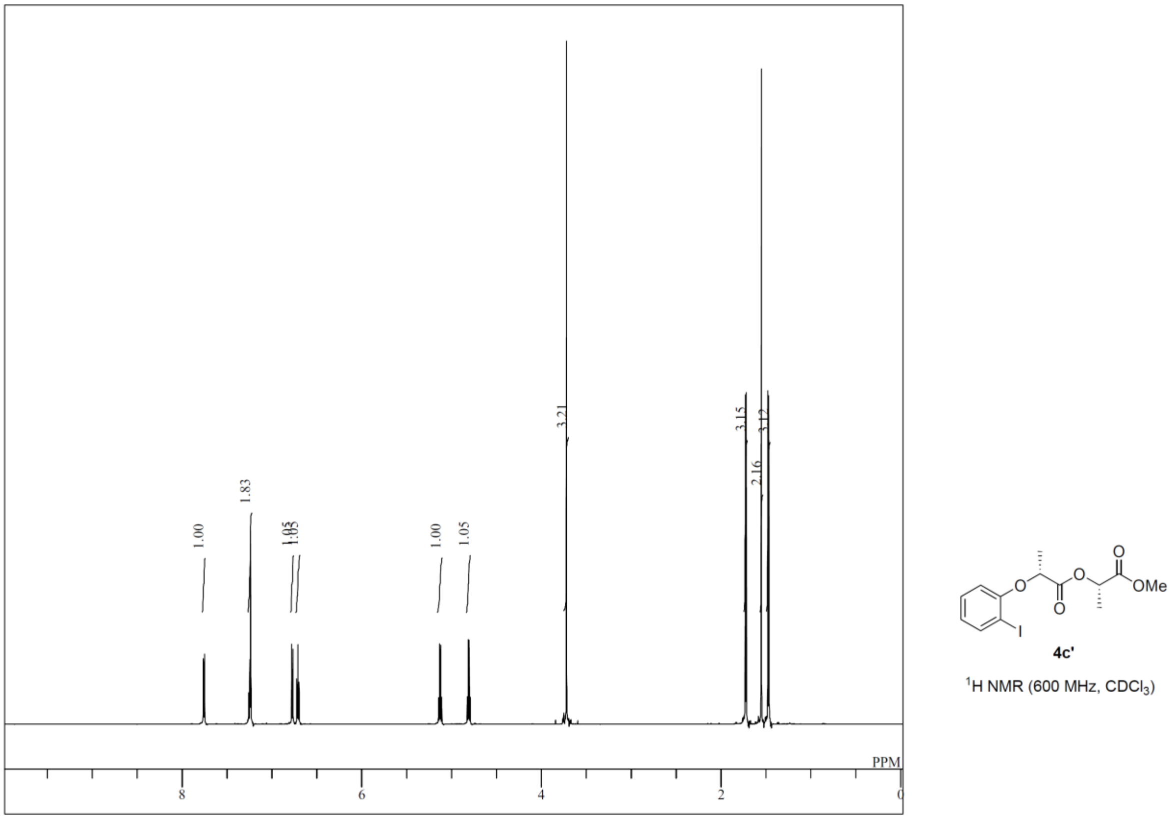

4c'

^{1}H NMR (600 MHz, $CDCl_3$)

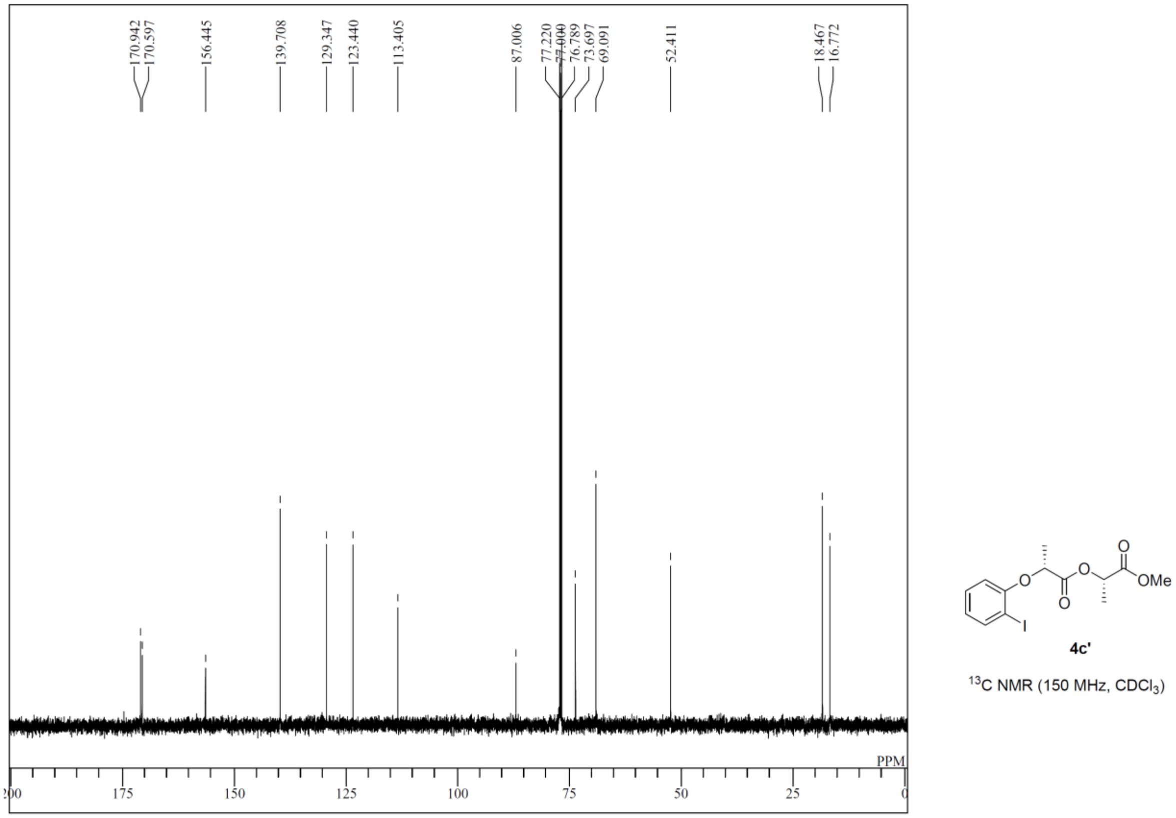

4c'

^{13}C NMR (150 MHz, $CDCl_3$)

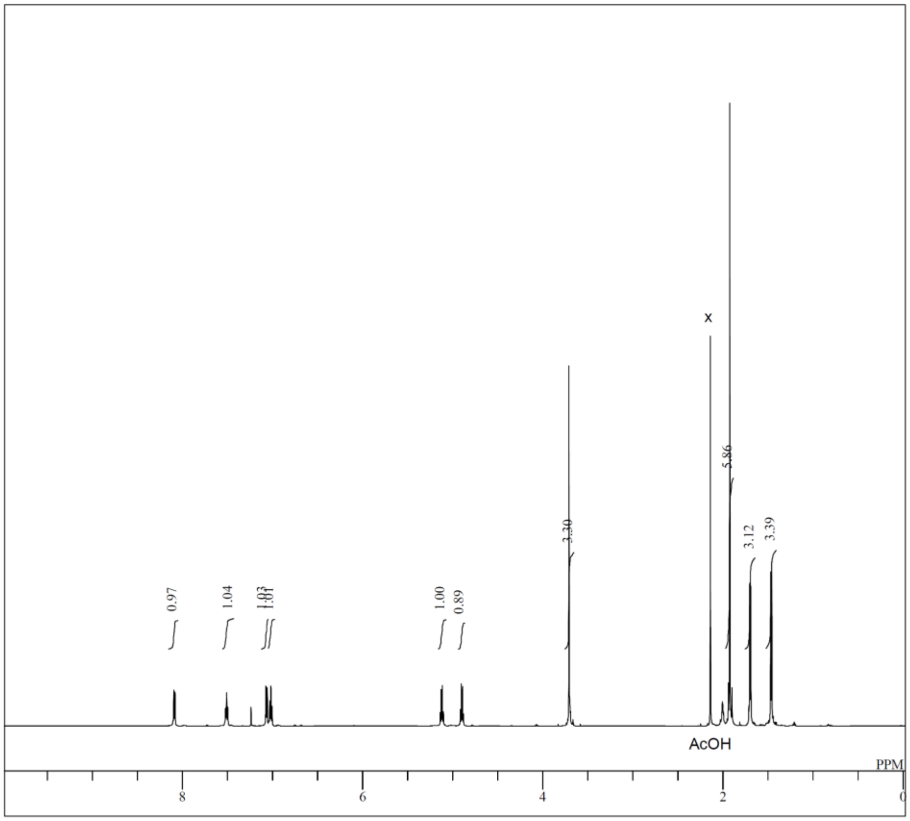

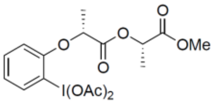

4c

^{1}H NMR (600 MHz, $CDCl_3$)

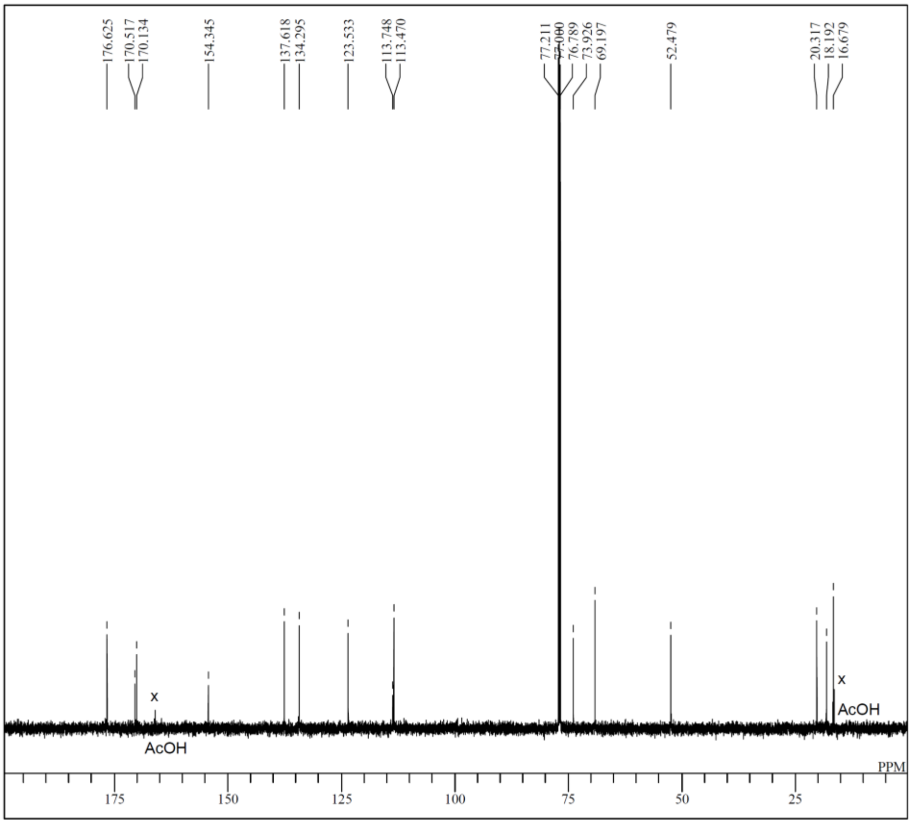

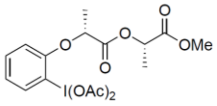

4c

^{13}C NMR (150 MHz, $CDCl_3$)

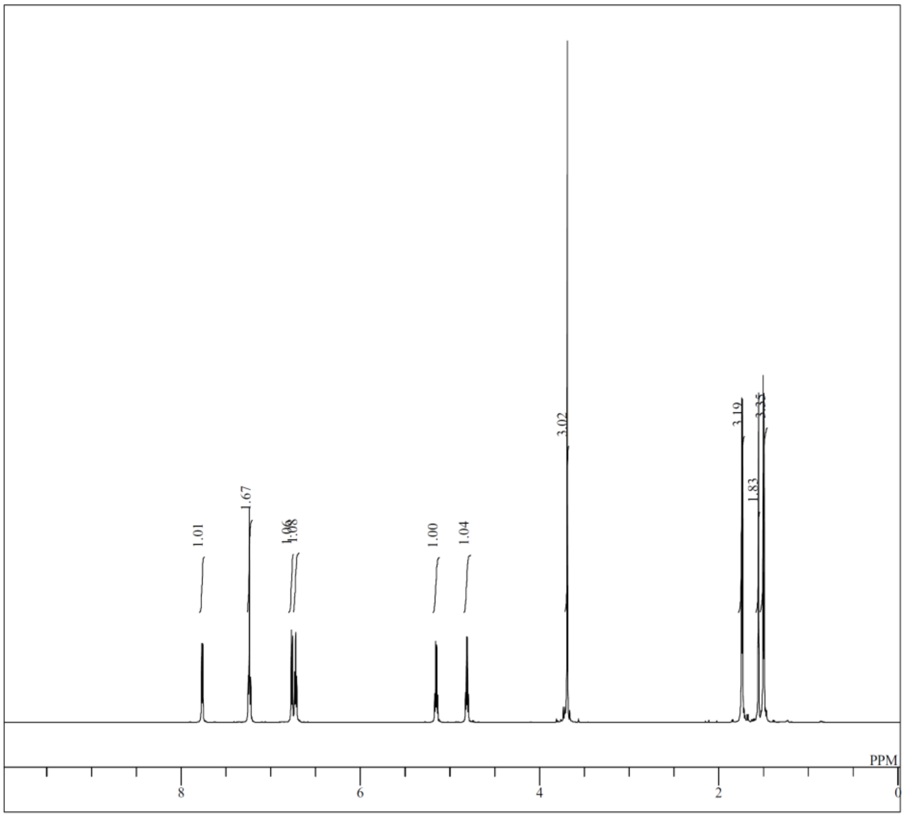

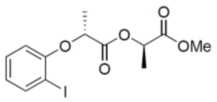

4d'

^{1}H NMR (600 MHz, $CDCl_3$)

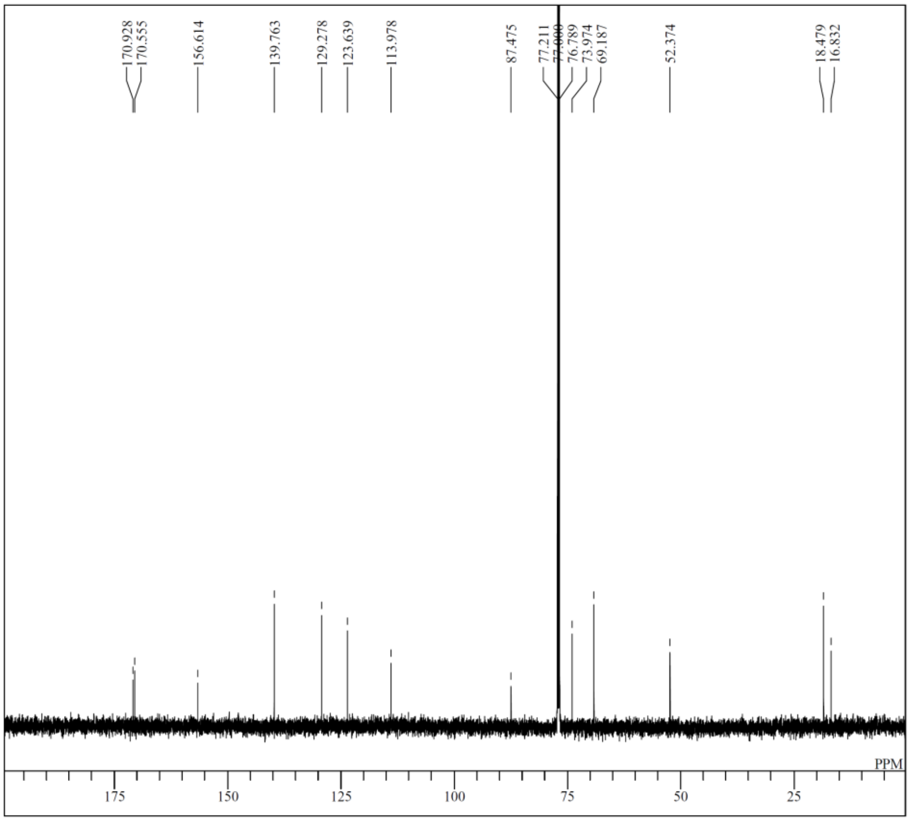

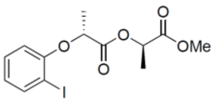

4d'

^{13}C NMR (150 MHz, $CDCl_3$)

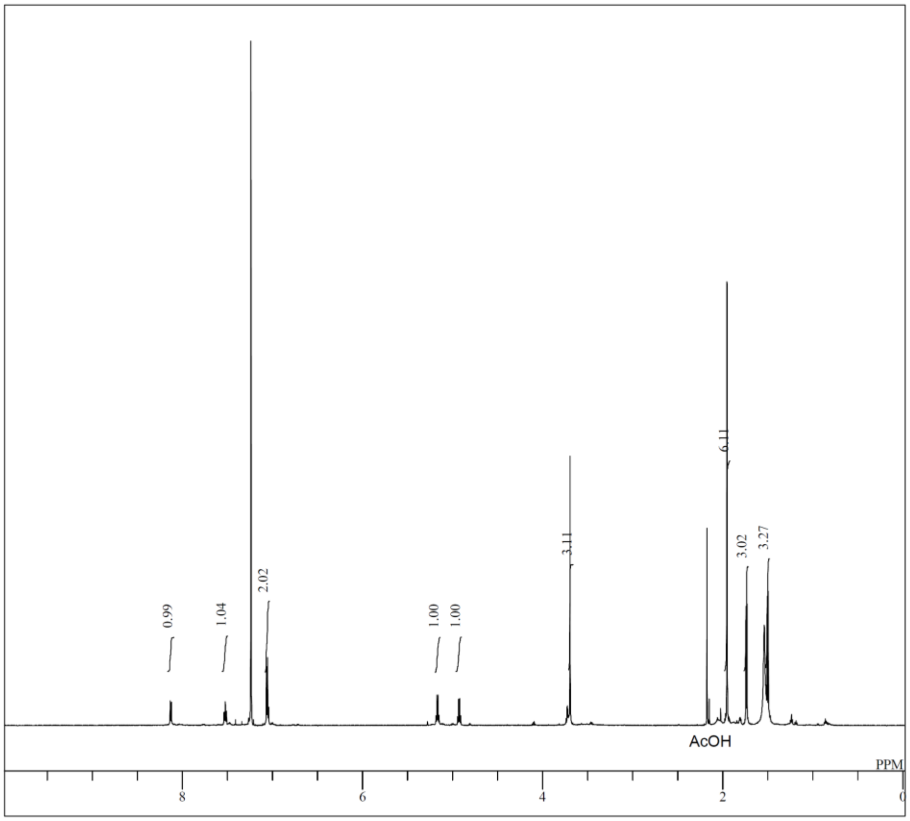

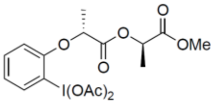

4d

^{1}H NMR (600 MHz, CDCl$_3$)

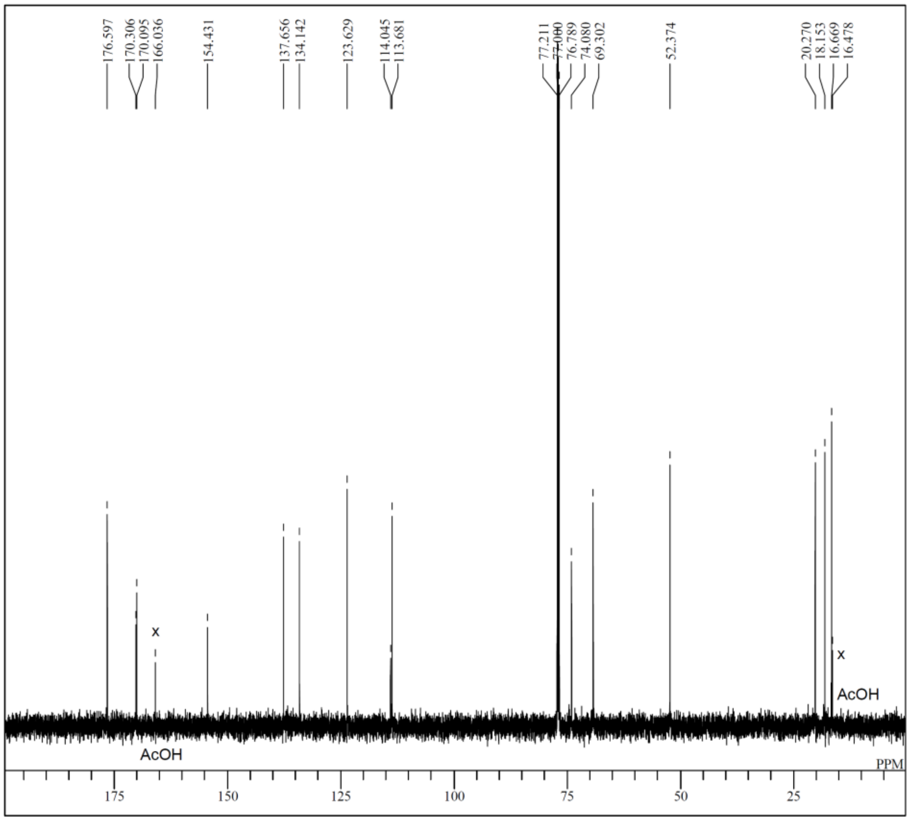

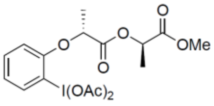

4d

^{13}C NMR (150 MHz, CDCl$_3$)

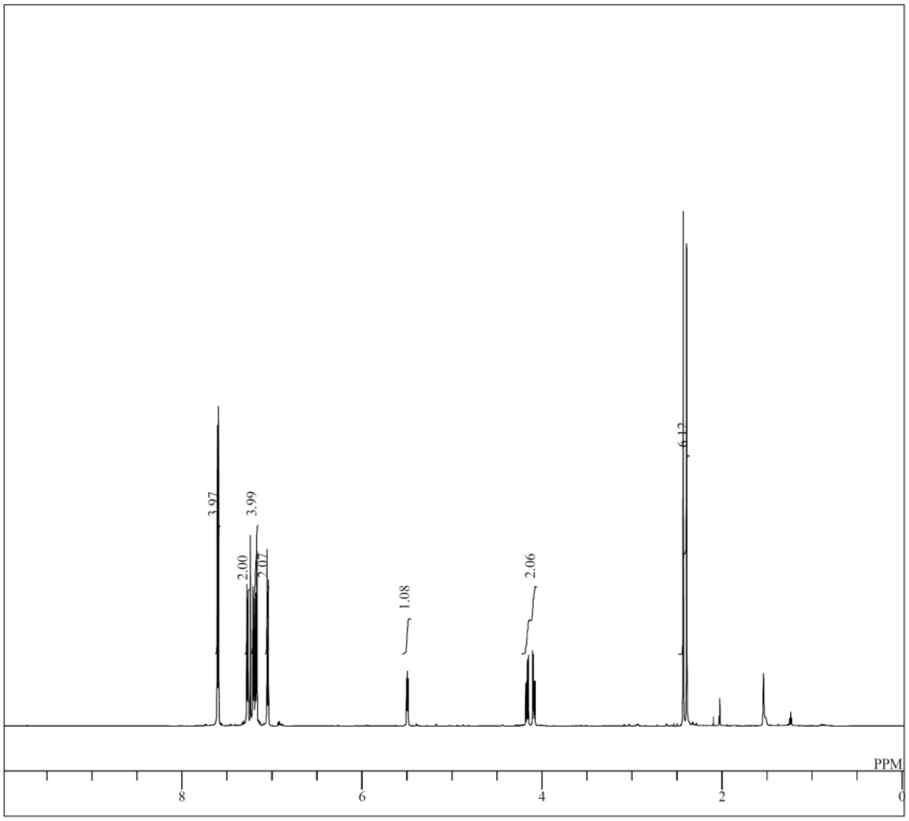

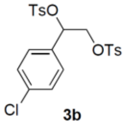

3b

^1H NMR (600 MHz, $CDCl_3$)

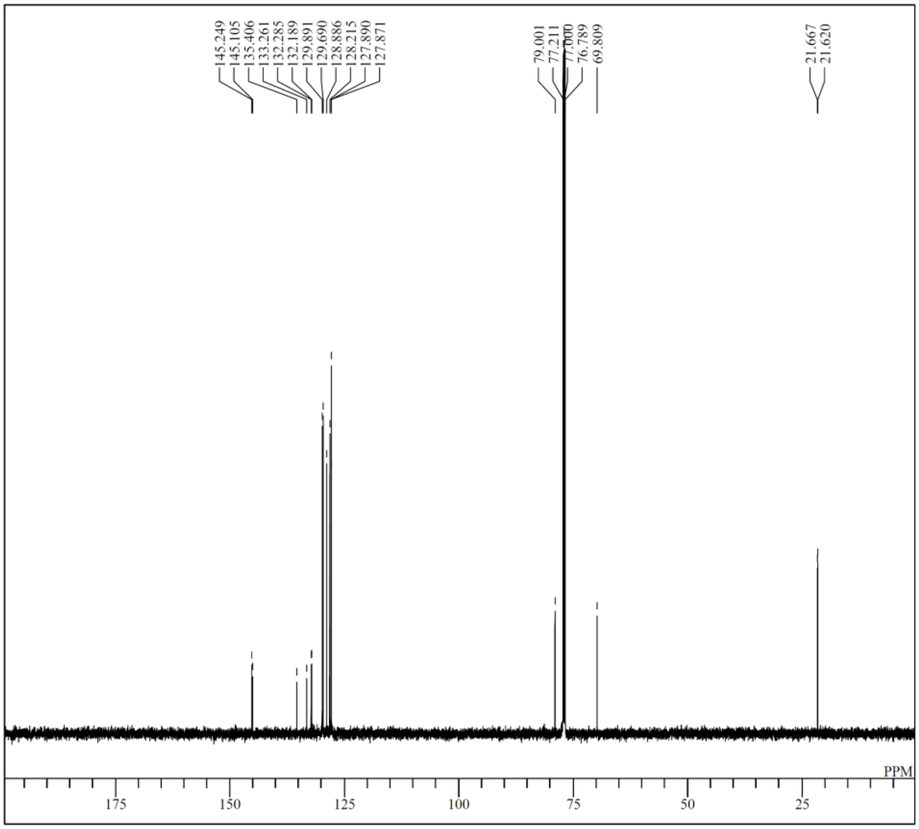

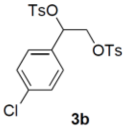

3b

^{13}C NMR (150 MHz, $CDCl_3$)

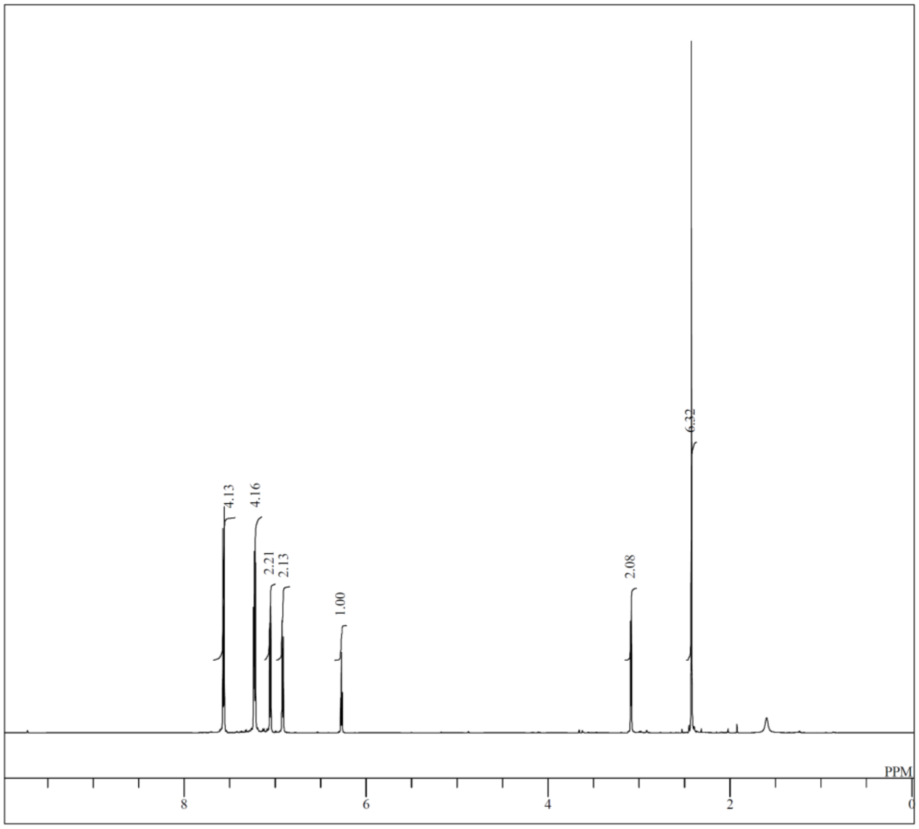

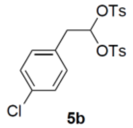

5b

^{1}H NMR (600 MHz, $CDCl_3$)

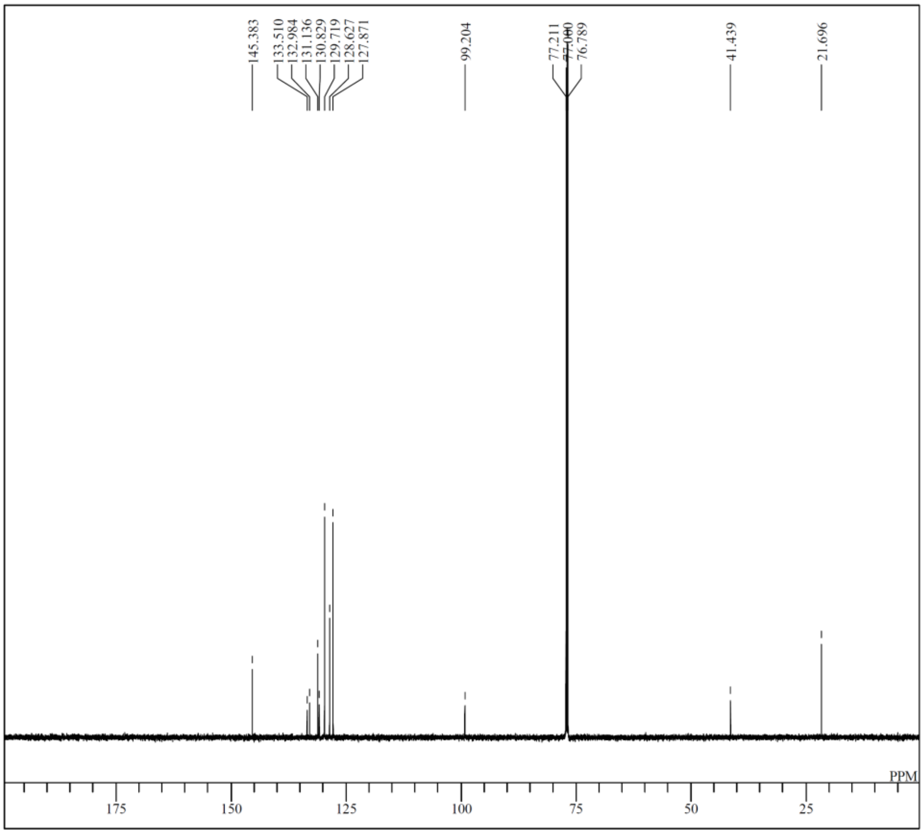

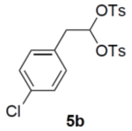

5b

^{13}C NMR (150 MHz, $CDCl_3$)

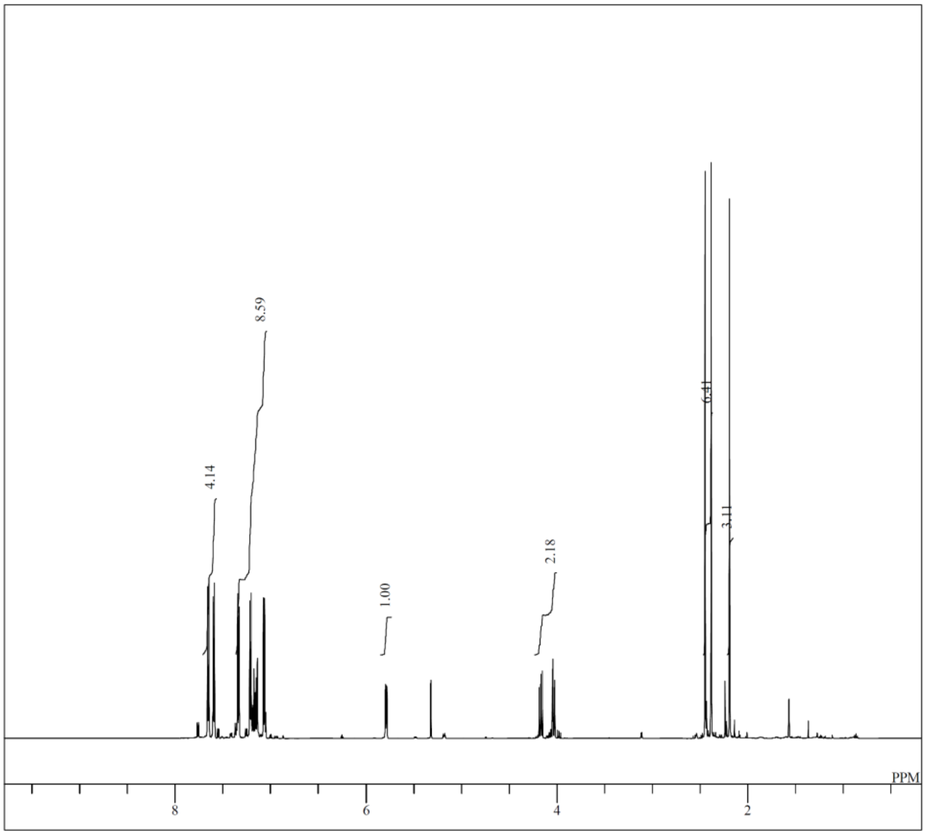

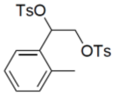

3c

^{1}H NMR (600 MHz, CD_2Cl_2)

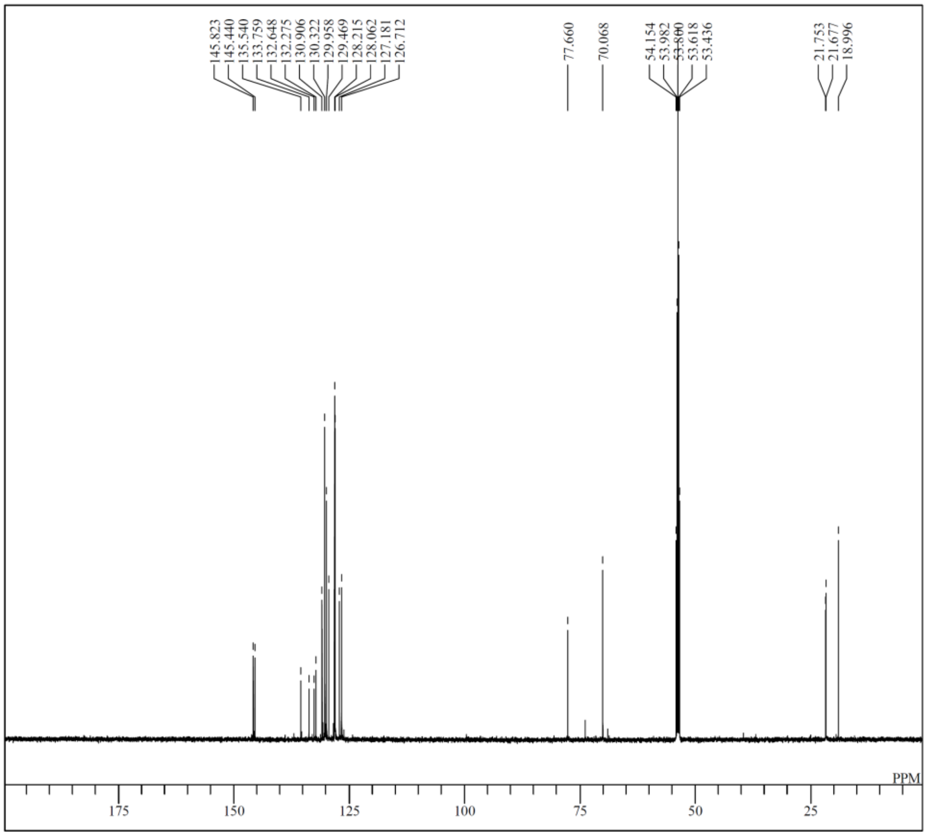

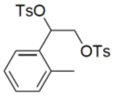

3c

^{13}C NMR (150 MHz, CD_2Cl_2)

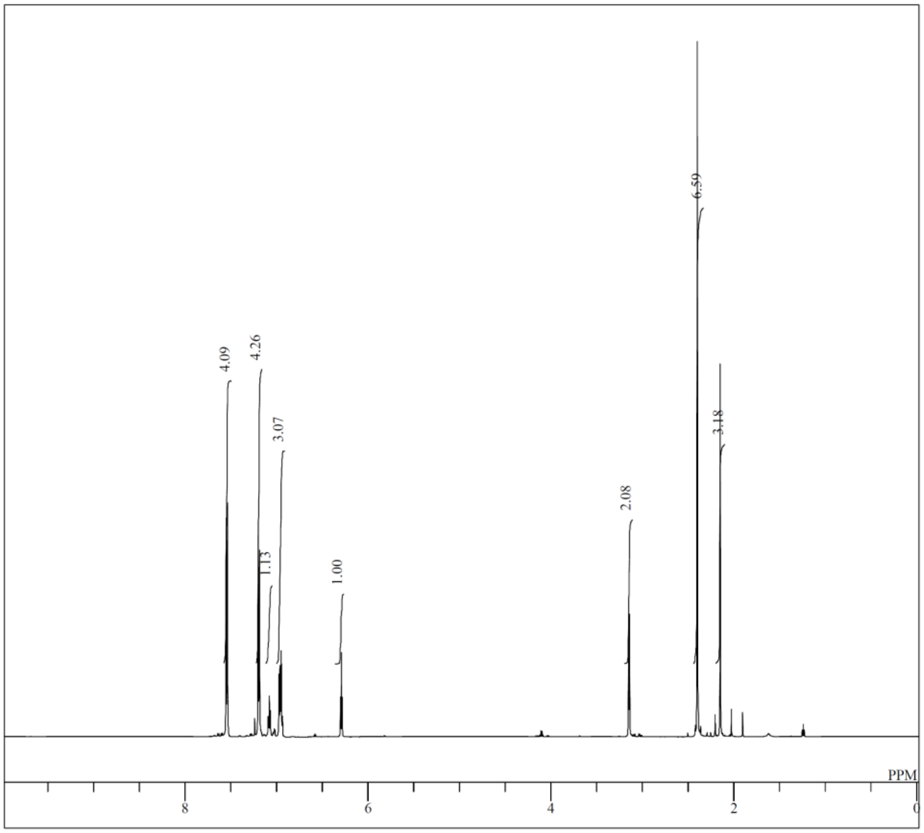

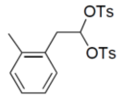

5c

^{1}H NMR (600 MHz, $CDCl_3$)

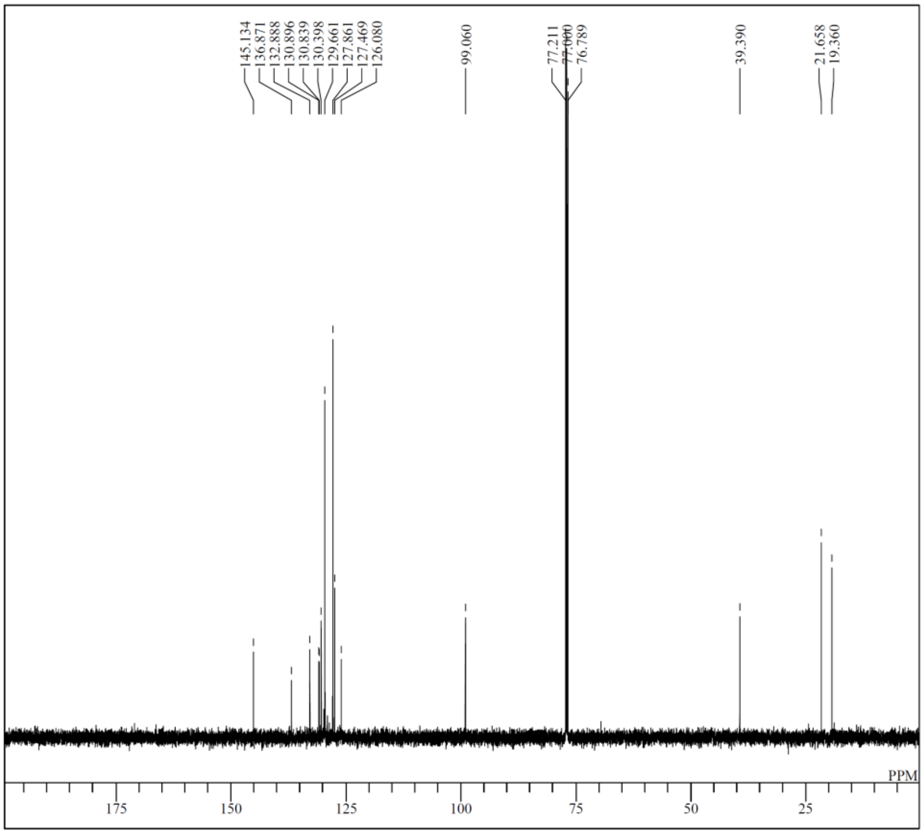

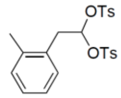

5c

^{13}C NMR (150 MHz, $CDCl_3$)
